# Spectrum of Heart Diseases in Children in a National Cardiac Referral Center Tanzania, Eastern Africa: A Six-Year Overview

**DOI:** 10.5334/gh.1342

**Published:** 2024-07-29

**Authors:** Naizihijwa G. Majani, Joëlle R. Koster, Zawadi E. Kalezi, Nuru Letara, Deogratias Nkya, Stella Mongela, Sulende Kubhoja, Godwin Sharau, Vivienne Mlawi, Diederick E. Grobbee, Martijn. G. Slieker, Pilly Chillo, Mohamed Janabi, Peter Kisenge

**Affiliations:** 1 Department of Pediatric Cardiology, Jakaya Kikwete Cardiac Institute, Dar es Salaam, Tanzania; 2 Julius Global Health, Julius Center for Health Sciences and Primary Care, Utrecht University, Utrecht, The Netherlands; 3 Faculty of Medicine, University of Utrecht, Utrecht, The Netherlands; 4 Department of Pediatrics, Muhimbili University of Health and Allied Sciences (MUHAS), Dar es Salaam, Tanzania; 5 Department of Pediatric Cardiac Surgery, Jakaya Kikwete Cardiac Institute, Dar es Salaam, Tanzania; 6 Department of Pediatric Cardiology, Wilhelmina Children’s Hospital, Utrecht, The Netherlands; 7 Department of Internal Medicine, Muhimbili University of Health and Allied Sciences (MUHAS), Dar es Salaam, Tanzania; 8 Department of Adult Cardiology, Jakaya Kikwete Cardiac Institute, Dar es Salaam, Tanzania

**Keywords:** pediatrics, epidemiology, congenital heart defects, Africa, Tanzania

## Abstract

**Background:**

While communicable diseases have long been the primary focus of healthcare in Africa, the rising impact of paediatric and congenital heart disease (CHD) cannot be overlooked. This research aimed to estimate the frequency and pattern of heart diseases in children who underwent their first echocardiography at a national cardiac referral hospital in Tanzania.

**Methods:**

A retrospective observational study was conducted on children aged 0 to 18 years referred for first-time cardiological evaluation from January 2017 to December 2022. Retrieval of social and echocardiogram data and descriptive analysis were performed.

**Results:**

There were 6,058 children with complete reports. Of these, 52.8% (3,198) had heart disease, of whom 2,559 (80%) had CHD, while (340/639; 53.2%) with acquired heart disease (AHD) had rheumatic heart disease (RHD). Children with CHD had a median age 1.0 years (IQR: 0.3–3.5) and were predominantly 51.2% male. Children with RHD had a median age 9.7 years (IQR: 3.2–13.8) with equal gender distribution. Shunt lesions were common in 1,487 (58.1%), mainly VSD 19.3%, PDA 19.1%, ASD 15.1%, and atrioventricular septal defect (AVSD) 4.6%. Pulmonary valve stenosis was in 97 (3.8%). Around 35% (718) had cyanotic CHD, with TOF being most common (13.3%), followed by double outlet right ventricle (DORV) (3.6%). Compared to global average truncus arteriosus was higher in 69 (2.3%) children. In contrast, TGA and hypoplastic left heart syndrome (HLHS) were lower than the estimated global average seen in 2.3% and 0.5% of the cases, respectively. Atresia of the right-side valves was more common (174 vs. 24), and approximately 40% of the patients referred for first-time echocardiographic evaluation required hospitalization.

**Conclusion:**

Congenital heart disease is the primary cause of heart disease in children presenting at a national referral hospital, surpassing RHD. With its distinct distribution pattern, acyanotic lesions are more frequent than cyanotic heart diseases. The observed late referral tendencies suggest improving the referral system, enhancing CHD awareness among healthcare professionals, and instituting nationwide screening programs.

## What was known about this topic?

1. Worldwide, congenital heart disease (CHD) is increasingly recognised as a childhood disease of public health importance.
2. There is a persistent burden of rheumatic heart disease (RHD) in developing countries.
3. Data are lacking in LMIC to inform policies and guide practice.
4. The distribution pattern of CHD may differ, driven by the healthcare landscape, genetic and environmental factors.
5. In developing countries, tetralogy of Fallot (TOF) is observed as the most common type of cyanotic heart disease. Ventricular septal defects (VSD), atrial septal defects (ASD), and patent ductus arteriosus (PDA) are the leading types of shunt lesions.

## What is new from this study?

1. Congenital Heart disease is the primary cause of referral for cardiac evaluation in children in this developing country setting, surpassing rheumatic heart disease.
2. The distribution pattern of certain types of CHD differs significantly from global estimates.
  - There is a recorded high prevalence of double outlet right ventricle, tricuspid atresia and truncus arteriosus, in addition to tetralogy of Fallot.
  - There were lower frequencies of transposition of great arteries (TGA) and hypoplastic left heart syndrome (HLHS) and so were atresia of the left-sided valves in general.
3. The inefficiency and inadequacy of the cardiac program are concerning in providing comprehensive cardiac care in this setting.

## Introduction

Healthcare efforts in Africa have traditionally focused on combating communicable diseases such as malaria, diarrhoea, pneumonia, malnutrition, HIV, and tuberculosis (1). As the magnitude of these communicable diseases decreases, to the credit of concerted international efforts, congenital heart disease (CHD) emerges as an important health concern (2,3). At a global level, CHD is recognised as the most common congenital anomaly (4). It is becoming the second leading cause of infant mortality in high-income countries (HIC) (6). Unfortunately, the exact prevalence of CHD in Africa is often unrecorded and underestimated (3,4,5). Nevertheless, in this region where rheumatic heart disease (RHD) is still endemic, there is a growing awareness of the significant impact of CHD (8,9,10,11,12,13).

Medical and surgical advancements enable infants born with CHD in North America and Europe to grow into healthy adults (7). Again, Africa faces a significant challenge, with the highest rate of unrepaired CHD cases; it is estimated that only 3% of children needing heart surgery in the continent receive it (9,10,31). The region’s limited access to specialised care, insufficient treatment facilities, and inadequate attention to cardiac illnesses in children are the primary reasons for this disparity (14,15,16,17,18,19,20,21). These regional inequalities in diagnosis and treatment extend to other paediatric heart conditions, including RHD (22). While RHD has been nearly eliminated in HICs, it still affects millions of children and adults in other regions, particularly sub-Saharan Africa (SSA) (15,22). In 2019, more than 33 million cases of RHD were reported globally, with over 90% residing in SSA (23).

Concerted efforts on multiple fronts are underway to alleviate the global burden and disparities in paediatric cardiac care and ensure better health outcomes for all individuals, regardless of their geographic location or socioeconomic status (18,20,21,24). This includes increasing awareness of risk factors, strengthening healthcare infrastructure, providing resources for early detection and management, and promoting preventive measures (20,21,25). Following these efforts, cardiovascular centres treating paediatric heart disease increasingly emerge in resource-limited settings, challenging the myth that such treatment is luxurious and excessively costly (16,26). In India and China, successful outcomes are achieved at lower costs than in the global North (27,28,29). However, these centres suffer from location challenges (far from most patients’ reach), leading to missed referrals, misdiagnoses, and delays in accessing specialised care due to transportation and financial limitations (16,28). Gathering local data is pivotal for addressing gaps and achieving the global goal of providing care to all children with heart disease (3,4,5).

In Eastern Africa, Tanzania, the Jakaya Kikwete Cardiac Institute (JKCI) has been the sole cardiac facility, serving a population of 60 million since 2015. It is equipped with two state-of-the-art catheterization laboratories and three operating theatres, including one dedicated to paediatric care. The institute has a team of well-trained paediatric cardiac surgeons, cardiologists, intensivists, cardiovascular nurses, and support staff. To date, the centre has performed over 2,500 surgeries on children and sees approximately 40 children daily in an outpatient clinic (unpublished local database). However, extensive data on patient demographics, the spectrum of cardiac diseases, and social characteristics at the JKCI are yet to be published. The present study aims to fill this knowledge gap and provide insights into paediatric cardiac care at a national referral hospital in a low-resource setting.

## Methods

### Study design

A single-centre retrospective study included patient data that had been documented in registration books/electronically between January 2017 and December 2022.

### Study setting

The Hospital Referral system in Tanzania operates at different levels. Level one is at the dispensary, level two at the health centre, level three at the district hospitals, level four at the regional hospital, level five at zonal hospitals, and the highest level at the national and specialised hospitals. The JKCI functions as a specialised national referral hospital. Patients will primarily be referred to JKCI from any of the 28 regional or six zonal hospitals. All other lower-level health facilities are advised against sending patients directly to JKCI.

### Study procedure

Cardiac consultation and echocardiography are performed by a paediatric cardiologist and well-trained and supervised paediatricians using two echocardiography machines: Siemens Logic 95 with 5S and 6S probes or a Vivid 9 Iq portable machine with 5H probes. The echocardiography included two-dimensional, colour, pulse wave, and continuous wave imaging. Routinely, a diagnosis is book recorded/entered into an online registry after a senior paediatric cardiologist’s confirmation. ICD-10 disease classification is used to document diagnosis. The data registry also provides a daily plan of care. During the hospital visit, a senior cardiologist who runs an outpatient clinic must develop a care plan indicating the need for surgery, hospitalization, and prescribed medications. Patients requiring surgical/catheterization intervention are listed and discussed during multidisciplinary meetings on Friday.

### Data collection

The JKCI transitioned to an online database for data recording in 2019. Data for the year 2015 were unavailable, and due to inaccuracies and incomplete entries in the registration books, data from 2016 and some from 2017 were excluded from the study. For 2017 and 2018, data were manually extracted from the registration book. The patient data included age, sex, type and severity of heart disease, and presence of extracardiac malformations, such as trisomy 21. Additionally, a conclusion regarding the need for an intervention, such as surgery, was drawn at the presentation time. From 2019 to 2022, data were retrieved from the online registry at JKCI by selecting new cases. The leading diagnosis was reported if more than one cardiac lesion was present. If additional data, including confirmation of age, was needed, patients were reached via telephone calls.

### Data sampling

The study included all patients aged between 0 and 18 who presented as new cases to the JKCI between 2017 and 2022, and were diagnosed with a cardiac condition upon echocardiography. Patients were excluded from the final analysis if information regarding age, visit type, and diagnosis was incomplete.

### Data analysis

The data were inspected for duplication, manually checked for inconsistency, inputted in SPSS, and analyzed by simple descriptive statistics. Quality control was ensured through the database’s introspection of electronic medical records and cross-checking with database reports.

## Results

**Figure 1:**
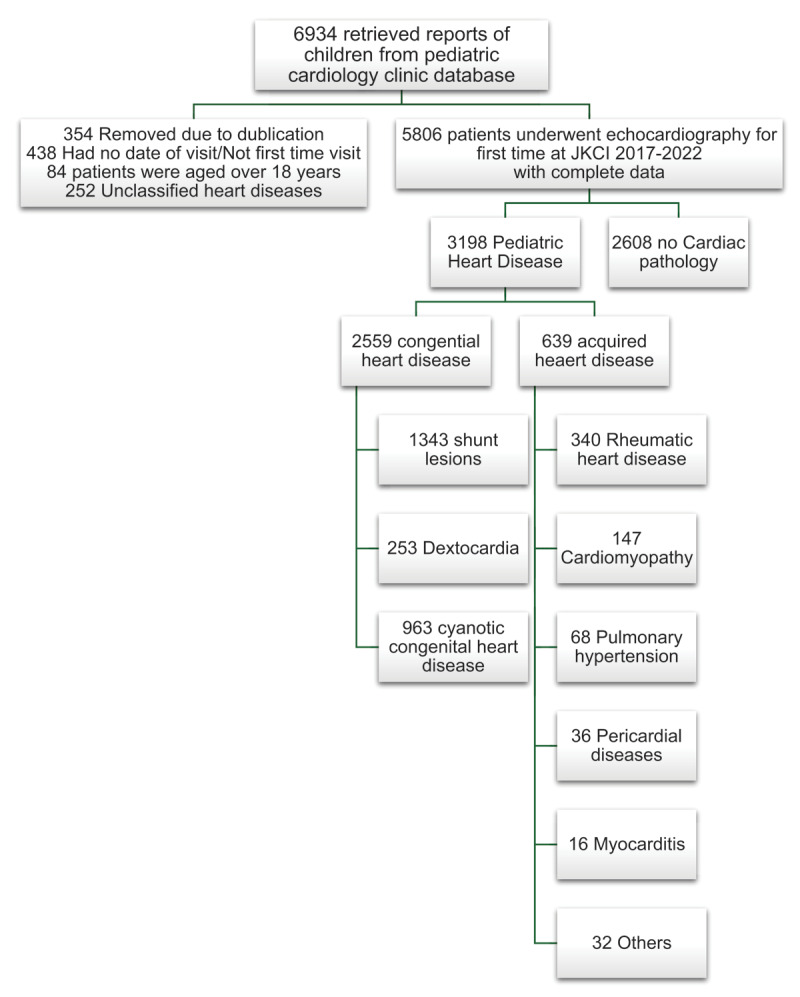
Flow chart of patients undergoing first Echocardiography at JKCI between 2017 to 2022.

Between January 2017 and December 2022, over six thousand (6,934) children were subjected to an echocardiogram for the first time at JKCI, of which 6,058 had a complete report and analyzed. More than half of the children, 52.8% (3,198), were diagnosed with heart disease, while the rest had normal echocardiograms (as shown in Figure 1).

Table 1 presents the demographic features of children with heart disease. The most common diagnosis was CHD, accounting for 80% (2,559/3,198). The median age of patients in this group was 1.0 years (IQR: 0.33–3.5), and 51.2% were male, with a median body weight of 7.0 kg (IQR: 5.0–12.0). Only 639 children were diagnosed with acquired heart disease (AHD), accounting for 19.7%; this group had a median age of 9.7 years (IQR: 3.2–13.8) and a weight of 21.0 kg (IQR: 11.0–32.6).

**Table 1:** Demographic Characteristics of Children with Heart Disease.


*VARIABLE*	*CONGENITAL HEART DISEASE (n = 2559)*	*ACQUIRED HEART DISEASE (n = 639)*	*TOTAL*

***Age in Yrs (Median; IQR)***	1.0 (0.33–3.5)	9.7 (3.2–13.8)	1.4 (0.4–5.8)

***Sex N(%)***			

***Female***	1250 (48.8%)	320 (50.1%)	1570 (49.1%)

***Male***	1309 (51.2%)	319 (49.9%)	1628 (50.9%)

***Body Weight in kg (Median; IQR),n = 2,403 CHD; n = 580 AHD***	7.0 (5.0–12.0)	21.0 (11.0–32.6)	8.0 (5.0–16.0)

***Height in cm (Median; IQR)***,*n* ***= 1,544 CHD; n = 352 AHD***	67 (57.0–89.0)	123.0 (86.3–144.0)	70.0 (59.0–103.0)


CHD = congenital heart disease; AHD = acquired heart disease.

### Congenital heart disease

Out of the 2,559 children diagnosed with CHD, the majority had acyanotic CHD (65%), while 893 (35%) of children presented with cyanotic CHD. Shunt lesions were observed in 1,487 (58.1%) children, primarily ventricular septal defect (VSD) at 19.3%, patent ductus arteriosus (PDA) at 19.1%, atrial septal defect (ASD) at 15.1%, and atrioventricular septal defect (AVSD) at 4.6%. Pulmonary valve stenosis was seen in 97 (3.8%) of children. For cyanotic heart disease, tetralogy of Fallot (TOF) was the most common at 13.3%, followed by double outlet right ventricle (DORV) at 3.6%. Truncus arteriosus was observed in 69 (2.3%) children, as was transposition of the great arteries (TGA) in 2.3% of the cases. Among those with congenital atresia of the cardiac valve, 87.8% were atresia of right-sided valves (pulmonary and tricuspid valves) at 6.8% of the total, compared to 0.9% for atresia of the left-sided valves (aorta and mitral valve). Additionally, congenital malformations of cardiac chambers (double inlet left/right ventricle) were observed in 233 (9.1%) of the total. Males made up the majority in all subgroups, averaging 51.1%, except for PDA, ASD, and AVSD, where females formed the majority at 59%, 54%, and 53%, respectively (see Table 2).

**Table 2:** Distribution of Various Congenital Heart Diseases by Gender and Year.


*LESION TYPES*	*GENDER DISTRIBUTION*				*AGE AT DIAGNOSIS*		*YEAR OF DIAGNOSIS*					
		
	MALE n 1,309	FEMALE n 1,242	TOTAL n 2,551	%	MEDIAN AGE	RANGE	2017	2018	2019	2020	2021	2022

*Ventricular septal defect*	279	213	492	19.2	1.0	16.5	112	54	83	58	101	84

*Patent ductus arteriosus*	200	286	486	19.0	0.9	17.3	67	45	70	69	135	100

*Atrial septal defect*	175	210	385	15.1	0.8	17.8	47	29	45	69	101	94

*Tetralogy of Fallot*	201	138	339	13.3	2.3	16.5	63	27	42	48	86	73

*Dextrocardia*	135	112	247	9.7	0.8	15.4	5	0	44	77	69	52

*Atrio ventricular septal defect*	56	63	119	4.7	0.6	17.1	29	15	15	4	26	30

*Pulmonary valve stenosis*	59	38	97	3.8	1.0	16.5	13	13	15	17	25	14

*Transposition of great arteries*	33	27	60	2.3	0.3	13.7	6	7	7	5	19	16

*Double outlet right ventricle*	49	44	93	3.6	1.0	17.7	11	7	16	13	23	23

*Persistent truncus arteriosus*	35	34	69	2.7	0.8	14.0	10	14	13	6	11	15

*Pulmonary valve atresia*	8	7	15	0.6	0.2	13.6	2	1	3	2	3	4

*Hypoplastic left heart syndrome*	6	7	13	0.1	1.4	6.7	0	0	0	3	7	4

*Tricuspid atresia (or stenosis)*	30	30	60	2.3	0.8	12.9	11	6	14	10	12	7

*Others*	43	33	76	2.9			5	5	11	12	18	23

***Total***							**381**	**223**	**378**	**393**	**636**	**539**


### Acquired heart disease

Among children with AHD, the majority (53.2%; 340/639) had rheumatic heart disease (RHD), followed by dilated cardiomyopathy 23.0% and pulmonary hypertension, 10.6%. The rate of pericardial diseases was 5.6%, and that of myocarditis was 2.5% (Table 3).

**Table 3:** Distribution of Acquired Head Disease.


*DISTRIBUTION OF ACQUIRED HEART DISEASES N 639*		

*TYPES*	FREQUENCY	PERCENTAGE

*Rheumatic heart disease*	340	53.2

*Dilated cardiomyopathy*	147	23.0

*Myocardidtis*	16	2.5

*Pericardial diseases*	36	5.6

*Pulmonary hypertension*	68	10.6

*Others*	32	5.0


The median age for the diagnosis of rheumatic heart disease was 11.0 years (IQR: 8.2–14.5), with females accounting for 48.2% of cases. The most common lesion types of RHD were mitral valve insufficiency and aortic valve insufficiency, both accounting for 26.8% of cases. Multiple valve disease was present in 21.8% of cases. Conversely, just 1.7% and 1.5% of cases, respectively, had mitral and aortic stenosis. The combination of mitral insufficiency and stenosis was present in only 2% of cases (Table 4).

**Table 4:** Distribution of Rheumatic Heart Disease by Valvular Type N 340.


*TYPE*	FREQUENCY	PERCENTAGE

*Mitral stenosis*	6	1.7

*Aortic stenosis*	5	1.5

*Combined mitral disease*	69	20.3

*Aortic insufficiency*	91	26.8

*Multiple valve disease*	74	21.8

*Mitral insufficiency*	91	26.8

*Tricuspid insufficiency*	4	1.2


### Plan of care

Out of the 2559 children diagnosed with CHD, 38% (990) required urgent attention during their first encounter in the cardiac facility. Of these children, 25% (249) required admission on the first day of echocardiography. The reasons for admission included medical stabilization (62%), surgery (29%), and catheterization procedures (8%). In addition, 69.1% (685/990) of these children needed further investigations but did not require admission. Children receiving medication as outpatients during their first visit were 5.7% (56).

Nearly 60% (1,524) of the children diagnosed with CHD had significant lesions that required intervention but were stable at their first visit and scheduled for follow-up. Only 1.8% (45) of the children with insignificant lesions were discharged from the clinic.

For those with RHD, nearly 60% had an established valvar disease, presenting with moderate/severe valvular damage, and needed surgical intervention on the first echocardiogram. However, they were in stable condition and received anti-failure treatment and Benzylpenicillin prophylaxis while waiting for planned surgery. Only 2% with severe RHD were too ill and needed urgent surgery, while 13.8% (88) were admitted on the first visit for medical stabilization and or/treatment for intercurrent illness while waiting for planned surgery (see Table 5).

**Table 5:** Plan of Care of Children with Heart Disease after First Echocardiography.


*PLAN*	*PLAN FOR CHILDREN WITH CONGENITAL HEART DISEASE*		*PLAN FOR CHILDREN WITH ACQUIRED HEART DISEASE*	
	
	FREQUENCY	PERCENT	FREQUENCY	PERCENTAGE

*Established diagnosis, need treatment*	1,524	59.6	375	58.7

*Admitted for catheterization*	21	0.8	0	0.0

*Admitted for surgery*	72	2.8	2	0.3

*Admitted for medical treatment*	156	6.1	88	13.8

*Discharge from clinic*	45	1.8	25	3.9

*Further investigations*	685	26.8	125	19.6

*Medications*	56	2.2	24	3.8


## Discussion

### Overall results

We conducted a retrospective review of children suspected of having heart disease who presented for the first time for echocardiography at the Jakaya Kikwete Cardiac Institute in Tanzania. This facility is the only one at the moment in the area offering comprehensive cardiac care to children and adults. Our research findings revealed a significant proportion, 80%, of CHD. In contrast, only 10.6% of patients were found to have RHD despite it being the leading cause of AHD. Our study’s CHD proportion is slightly higher than that of a comparable study conducted in Tanzania by Zuechner et al., which reported a proportion of 75%, but it is not surprising (32). Evidence from various reports in Africa indicates that CHD is becoming a more prevalent cause of childhood heart disease, surpassing RHD, driven by improved awareness, improved diagnostic tools, and enhanced socioeconomic conditions in the region (33,34,35,36,37,38,39,40,41).

### Congenital heart disease

Expectedly, most, 65%, of the children with CHD in our cohort had acyanotic heart diseases, with VSD being the most common subtype, at a rate of 19%. Noteworthy, the frequency of VSD in African studies is lower (16–29%) than the estimated global average of 35% (5, 35). In Sudan, the VSD rate was 16.6% in a cohort of 596 patients; in Nigeria, it was 25%; in South Africa, 20%; in Botswana, 29%; and in Uganda, 27.2% out of 3526 patients (33,34,35,36,37). Similarly, a comparable study conducted in Tanzania from 2009 to 2016 indicated that the frequency of VSD was 26.1%, and a recent meta-analysis showed that the proportion of VSD in CHD in East Africa is 29.9% (32,42). This difference in frequency distribution between regions is because CHD is diagnosed early in the North. Thus, most shunt lesions are detected, whereas, in the global South, limited financial resources and healthcare-seeking behavior may result in only children with symptoms seeking medical attention (10,13,14). As a result, many shunts may naturally close when they see a doctor or die before a diagnosis is made (15,16,17,18).

A significant difference in the distribution pattern was also observed in atrioventricular septal defect (AVSD), the most severe form of shunt lesions. In our study, the frequency of AVSD was 5%, similar to findings from other African studies. In the PROTEA registry in South Africa, AVSD was recorded at 7%, whereas in Sudan, it was 9%, Nigeria, 6%, and Uganda, 8% (33,34,35,37). Inarguably, our study’s distribution pattern of shunt lesions could indicate a lack of access to early detection, lack of prenatal screening, infrequencies of pregnancy termination, and selective detection of symptomatic lesions. However, further investigation is required to explore whether genetic and environmental factors contribute to this trend. Nevertheless, our findings confirm that most CHDs are relatively simple and amenable to surgical intervention (3,4,5,43,44).

Nearly a third of our patients with CHD presented with cyanotic heart disease. Moreover, the frequency of certain types of cyanotic heart diseases appeared higher in our cohort than in the global estimates. Specifically, we observed a greater occurrence of tetralogy of Fallot (13%), double outlet right ventricle (4%) and truncus arteriosus (2%), significantly higher than the global averages of 5%, 1.3% and 1%, respectively (5). On the other hand, we found a lower prevalence of transposition of the great arteries (TGA) (2% vs. 5%) and hypoplastic left heart syndrome (HLHS) (0.5% vs. 2.5%). Of interest, we observed a high frequency of tricuspid atresia (3%), three times the global estimate and higher than reported in South Africa (1%) and Uganda (1.8%) (35,37).

These differences in the distribution pattern of cyanotic heart diseases in our group may be due to differences in detection abilities. Since there is no systematic screening for CHD in the region, we assume that the observed pattern is mainly due to survival. The most severe cases may not have been detected at birth and could have resulted in death before detection. DORV, AVSD, and TOF are highly symptomatic, and tricuspid atresia has a balanced circulation nature, which allows for detection and, thus, the observed trends. Meanwhile, HLHS and TGA are lethal and, therefore, escape detection. It is important to note that the cyanotic CHD cases identified in our cohort are variations of the typical lesions that can lead to survival, while the more severe cases that result in death were not identified. This underscores the fact that serious forms of congenital heart disease (CHD), especially CCHD, are often overlooked or don’t make it to specialised medical centres in developing countries due to limited screening of newborns and prenatal detection practices in these areas (12,13,14,15,16,17,18). However, we must recognise the differences in environmental exposure and genetic variability between the two hemispheres. Environmental factors, such as calcium deficiency and particulate matter, are believed to play a role in the incidence of some CHD. Targeted research and interventions are needed to better understand and control these risk factors (45,46,47,48,49).

Lastly, we observed a high number of patients, 247, presenting with dextrocardia in the CHD group, accounting for 10% of the total cases. This is ten times higher than the global average and the 0.4% recorded in the Eastern Africa series (37). Dextrocardia is a rare disorder of cardiac laterality with an unknown cause. It is thought to have a genetic influence based on its association with other genetic syndromes, such as Kartegener syndrome, and familial clusters. In population studies, its incidence is estimated to be 1 in 12,000 pregnancies (50,51). Given the high rate of occurrence in our study, further investigation, including genetic determination, is necessary.

In terms of demographic characteristics, our CHD cohort had ages ranging from 0.35 to 3.5 years, older than those of the global North, whose children are mostly diagnosed in utero or soon after birth (3,4,5). These findings were not unexpected and attributed to the lack of a coordinated screening program in our setting. With respect to gender distribution, it is well known that males are more susceptible to complex congenital heart disease, while females are more likely to have shunt lesions (52). Therefore, the predominance of males in our cohort, given that the majority had shunt lesions, was surprising. However, it is important to note that gender distribution has shown different patterns in different groups, likely due to the dominance of specific lesions (52,53).

### Burden of RHD and other acquired heart diseases

As expected, RHD was the most common AHD in our cohort study, accounting for 53.2% of the cases. This was followed by cardiomyopathies and pulmonary hypertension.

Globally, RHD is the primary cause of AHD in children and young adults, with a current estimated prevalence rate of 26.1% (23). However, a recent meta-analysis in East Africa suggests a decreasing trend of RHD in the region, with a reported rate of 14.6%, which is lower than the global estimate (55). This positive trend has been attributed to improvements in health services, better living conditions, and empowered communities (22,23,30,54). Despite this, RHD remains common in many LMIC countries, particularly rural areas (23). Our RHD’s proportion rate of 10.6% should be interpreted cautiously, as we lack a national RHD control program. Furthermore, as it is a disease of poverty, and with only one cardiac centre in the area, it is possible that many children with RHD cannot afford to travel for care (56). Thus, concerted efforts are required to identify high-risk areas and strengthen surveillance measures in Tanzania.

Unsurprisingly, dilated cardiomyopathy was the second most common cause of AHD in our cohort. Other studies, too, have recorded dilated cardiomyopathy as the second most common AHD in children (57,58,59). It is the most common cause of transplant need in the global North, but its true burden in the global South is still lacking (60). However, having been associated with malnutrition and infectious disease, the burden is suspected to be substantial (22).

Our third most prevalent AHD, pulmonary hypertension, is increasingly recognised as an important cause of childhood mortality and morbidity; in children, it stems from the highest number of unrepaired CHD and RHD (61,62). Since our cohort records a high rate of pulmonary hypertension, close follow-up is essential.

### Plan of care

Finally, our research revealed a concerning issue in the referral process for children with heart conditions. Nearly 60% of the referred children had no cardiac diagnosis, indicating an inaccurate referral. Among those with a cardiac diagnosis, 40% had to be hospitalised immediately upon arrival, suggesting delayed referral. The issue of inefficient referrals for children with heart conditions affects both high- and low-income countries (63,64,65). The root causes of this problem include a flawed referral system, a lack of trust in lower health facilities, and challenges that primary healthcare workers (PHWs) face in determining which patients to refer (66,67,68). In LMICs, families face additional referral delays due to limited accessibility to referral facilities, lack of transportation, and financial limitations (56).

Late diagnosis not only puts children at risk of additional health complications but also leads to poor surgical outcomes (28). In emerging cardiac programs like JKCI, inefficient referrals force the team to operate urgently and uncoordinatedly, jeopardizing output. To address these issues, PHWs require comprehensive training, standardised screening tools, and access to telemedicine services (15,24,27). Collaborating with specialised cardiac centres and establishing clear referral pathways can ensure timely diagnosis and treatment (20,21). Subsequently, implementing quality assurance programs to monitor and evaluate the performance of PHWs will help improve their diagnostic skills (19,73). Where feasible, exploring intelligent auscultation and using clinical scores can improve referrals and prevent overcrowding; indeed, implementing intelligent auscultation in resource-limited settings has been an area of active research in recent years (68,69,70,71,72).

## Limitations and Future Research Recommendations

This study has certain limitations. First, it being a hospital-based study introduces selection bias, leaving out children with subclinical symptoms or no access. Moreover, referral decisions were made by the referring doctors, potentially leading to a focus on patients with more severe symptoms and potentially overlooking asymptomatic patients. For these reasons, patients may not fully represent the actual situation in the broader community. However, it is worth noting that this particular centre serves as the sole referral centre for children with heart disease in the country. This study, therefore, provides insight into the burden and distribution of childhood heart disease in Tanzania.

Secondly, the retrospective nature of the study was limited by missing data. Therefore, we propose a prospective study and registry to better understand the nature of heart diseases affecting children in the country. Such initiatives would aid in identifying modifiable risk factors for prevention and shed light on ways to improve care delivery.

Despite these limitations, this study has provided valuable insights into the country’s healthcare system challenges for children with heart disease.

## Summary

Heart disease poses a significant challenge for children in Tanzania. Despite the government’s efforts to make services available and provide super-specialised training in cardiovascular-related disciplines for doctors, nurses, and allied health care at the national level, several challenges must be overcome. One significant challenge is the inefficiency of referrals, which may compromise outcomes, overburden healthcare resources, and create inefficiency in cardiac care provision. An improved referral system for childhood heart disease must be established to address this challenge. One solution is training PHCW providers and implementing clinical aid decision-making tools at primary health facilities to ensure appropriate and timely referrals. Additionally, policies should be enacted to include auscultation for every child attending well-baby clinics and while managing sick children at all levels in the country. Increasing the number of facilities available to manage childhood heart disease is also crucial.

In terms of heart disease distribution in the country, CHD is more prevalent than RHD at presentation. While this may indicate the disappearance of RHD, a lack of an RHD control program may suggest a lack of access; an RHD control program, therefore, is desirable. On the other hand, the distinct distribution patterns in CHD subtypes show the need for a comprehensive CHD registry to better understand the nature of CHD and its associated factors in this setting. Establishing a CHD screening program at birth for early detection is also necessary, and the rate of observed dilated cardiomyopathy and pulmonary hypertension requires close attention. Thus, implementing these measures will be essential in providing better care and improving outcomes for children with heart disease in Tanzania.

## Conclusions

Congenital heart disease (CHD) is the primary cause of heart disease in children presenting at the JKCI, surpassing rheumatic heart disease (RHD). Shunt lesions are more common, but the distribution of the CHD subtype differs significantly from global estimates. Notedly, the rate of late and inappropriate referrals to JKCI poses a significant challenge and may jeopardise patient outcomes. To address this, it is crucial for the health system to improve referrals, enhance awareness among healthcare professionals, and institutionalise screening programs.

## Data Accessibility Statement

The authors will make the raw data supporting this article’s conclusions available without undue reservation upon request to the corresponding author.

## Abbreviations

AHD – acquired heart disease

CHD – congenital heart disease

RHD – rheumatic heart disease

JKCI – Jakaya Kikwete Cardiac Institute

CCHD – critical congenital heart disease

PHCW – primary health care workers

HIC – high-income countries

LMIC – low- and middle-income countries
